# Body mass index in type 2 spinal muscular atrophy: a longitudinal study

**DOI:** 10.1007/s00431-021-04325-3

**Published:** 2022-01-19

**Authors:** Gloria Ferrantini, Giorgia Coratti, Roberta Onesimo, Simona Lucibello, Sarah Bompard, Ida Turrini, Graziamaria Cicala, Michela Caprarelli, Maria Carmela Pera, Chiara Bravetti, Beatrice Berti, Valentina Giorgio, Claudio Bruno, Noemi Brolatti, Chiara Panicucci, Adele D’Amico, Antonella Longo, Chiara Leoni, Valeria A. Sansone, Emilio Albamonte, Sonia Messina, Maria Sframeli, Enrico Bertini, Marika Pane, Eugenio Mercuri, the Italian ISMAC study group

**Affiliations:** 1 Pediatric Neurology, Università Cattolica del Sacro Cuore, Rome, Italy; 2 Centro Clinico Nemo, Fondazione Policlinico Universitario Agostino Gemelli IRCCS, Rome, Italy; 3 Pediatric Unit, Fondazione Policlinico Universitario A. Gemelli IRCCS, Rome, Italy; 4 Center of Experimental and Translational Myology, IRCCS Istituto Giannina Gaslini, Genoa, Italy; 5 Unit of Neuromuscular and Neurodegenerative Disorders, Department of Neurosciences, IRCCS Bambino Gesù Children’s Hospital, Rome, Italy; 6 Neurorehabilitation Unit, University of Milan, Neuromuscular Omnicentre Clinical Center, Niguarda Hospital, Milan, Italy; 7 Department of Clinical and Experimental Medicine and Centro Clinico Nemo Sud, University of Messina, Messina, Italy

**Keywords:** Neonate, Children, Spinal muscular atrophy, Body mass index, Nutritional status

## Abstract

The aim of this retrospective study was to review body mass index (BMI) in a large cohort of Italian pediatric type 2 spinal muscular atrophy (SMA) patients, aged between 0 and 20 years and to establish possible differences in relation to a number of variables such as ventilation, motor function, and survival motor neuron 2 gene copies. Cross-sectional data were collected from 102 patients for a total of 344 visits. Standard growth charts for height and weight were used as reference, with age adjusted BMI calculated using the Center for Disease and Prevention Children’s BMI Tool. In the 344 visits, weight ranged between 3.90 and 83 kg, and the BMI between 8.4 and 31.6 with a BMI/age *z*-scores <  − 2SD present in 28% and BMI/age *z*-scores >  + 2SD in 9% of the measurements. The BMI/age *z*-scores were relatively stable < 5 years of age with an increasing number of patients <  − 2SD after the age of 5, and a wider range of BMI/age *z*-scores after the age of 13. A difference on the BMI/age *z*-scores was found among the different age subgroups (< 5, 5–12, ≥ 13 years). A multivariate analysis in 58 patients with longitudinal assessments showed that baseline BMI/age *z*-scores and gender were significantly contributing to the changes while other variables were not.

*Conclusion*: Our results confirm that careful surveillance of weight and BMI/age *z*-scores is needed in type 2 SMA. Further studies, including assessments of chewing and swallowing and of lean/fat body mass, will help to better understand the possible mechanisms underlying weight issues.

**Table:** 

**What is Known:** *• Feeding difficulties have been reported in a few studies and were invariably found in patients with type 1 SMA.* *• Type 2 SMA patients often have low BMI with a relevant number of patients requiring tube feeding.*	
**What is New:** *• Reduction in BMI/age z-score overtime appeared to depend on baseline BMI/age z-score and gender.* *• Patients with a low BMI/age z-score were at higher risk of developing further reduction.*

**Supplementary Information:**

The online version contains supplementary material available at 10.1007/s00431-021-04325-3.

## Introduction

Spinal muscular atrophy (SMA) is an autosomal recessive disease caused by mutations in the survival motor neuron 1 (*SMN*1) gene [1]. The recent advent of therapeutic options has changed the progression of the disease [2–5]. After successful clinical trials leading to regulatory approval, several papers have reported real world data [6–10] reporting efficacy and safety in large cohorts including patients of age and severity that had not been included in the clinical trials. The real-world data have highlighted the need to have detailed information on several aspects of natural history for comparison with the treated patients.

Most natural history studies have focused on motor and respiratory function [11–19]. Feeding and nutritional difficulties have been reported in a few studies [18–30] and were invariably found in patients with type 1 SMA. Less has been reported in type 2 patients, with a limited number of studies showing heterogeneous results [20–31].

Particular attention has been paid to weight and body mass index (BMI). Type 2 SMA patients often have low BMI with a relevant number of patients requiring tube feeding [31, 32]. A recent paper reporting questionnaires in two cohorts from different countries suggest that some variability is present and this may be partly due to different cultural backgrounds [30].

We report our experience in a large cohort of Italian pediatric type 2 SMA patients in whom anthropometric data were available for a retrospective analysis of BMI/age *z*-scores at different ages. We also aimed to establish possible differences in BMI/age *z*-scores in relation to a number of variables such as respiratory support, motor function, and *SMN2* copies.

## Material and methods

We included all available data from type 2 patients aged between birth and 20 years with a genetically confirmed diagnosis of SMA from 5 clinical referral centers in Italy (Bambino Gesù Hospital, Rome; Niguarda Hospital, Milan; University of Messina, Messina; Istituto Gaslini, Genoa and Policlinico Gemelli, Rome).

Data from visits of patients in treatment with investigational drug or approved disease-modifying treatments were not included. From June 2018 data had been collected using the recently developed international SMA registry [33].

In all centers, as part of the clinical routine, anthropometric measures (weight, height/length) were obtained by standardized procedures. Infants were weighed on a standard infant scale, while in older children who were too large for this scale, weight of the subject was obtained by using a chair scale as they could not be assessed while standing.

In children younger than 24 months, supine length was measured by infantometer. In those older than 24 months, since sitters and non-sitter patients often develop contractures, we used ulnar length and derived the total length as previously described [34, 35]. These methods of conversion have been widely used in SMA and non-SMA disease from 24 month of age to adulthood in both clinical trials (NCT02908685) and clinical settings [17, 36, 37].

BMI was defined as body weight divided by the square of recumbent length in meters. Sex-specific weight, length, and BMI/age *z*-scores were derived using the 2006 World Health Organization growth charts for patient < 2 years old and the 2000 Center for Disease Control and Prevention growth charts for older patients [38–42]. All children with at least one weight recorded in clinical charts were included in the study. Visits with missing data on height were excluded from the analysis. Statistical analysis (frequency table, *t*-test, and chi-square test) of missing data is available in supplementary file 1.

Nutritional status of the patients was determined by the calculus of BMI/age *z*-scores. A child whose BMI/age *z*-score was <  − 2SD was considered as underweight, conversely, who had a BMI/age *z*-score >  + 2SD was classified as overweight.

### Statistical analysis

Demographic and clinical characteristics were summarized using frequencies (percentage) for categorical variables and mean (standard deviation (SD)) or median (range) for continuous variables. Differences between the whole cohort and the longitudinal cohort were assessed with *U* Mann–Whitney test for age and BMI *z*-score at 1st visit and chi-square test to analyze distribution of patients among gender, SMN2 copies, functional status at 1st visit, and G-tube at 1st visit.

Kruskal–Wallis with Dunn-Bonferroni correction was used to compare differences in BMI/age *z*-score among age subgroups, using predefined cutoff points for age identified in previous studies on the basis of slope of functional deterioration (< 5, 5–12, ≥ 13 years) [8, 11, 15, 43, 44]. Chi-square test was used to analyze assessments’ distribution of patients with BMI/age below − 2SD, within the − 2 and 2SD, or above 2SD. The chi-square test was applied subdividing the population by gender, functional status (non-sitter/sitter), or ventilatory status (spontaneous breathing/non-invasive ventilation) at 1st visit. Functional status was determined by the ability of the patient to sit by himself for at least 3 s (sitter) or not (non-sitter), as used in outcome measures such as the HFMSE and in recent literature [45, 46].

A mixed model was used to estimate the effects of different variables on weight. The model was set up with age, gender, *SMN2* copy number, functional status, non-invasive ventilation (NIV), nutritional status (oral intake solid, oral intake semi-solid, G-tube), scoliosis surgery, BMI/age *z*-score at baseline as fixed effects, and patient as random effect. To make inferences about mean slopes of weight, the model was expanded by including appropriate main effect and interaction terms in the model.

For all the analyses the *p*-value was set at *p* < 0.05.

The study was approved by the ethics committee in each center. As part of this study all participants and/or their legal representatives provide written informed consent for use of the prospective and retrospective clinical data for academic purposes.

## Results

### Cross-sectional analysis

Data were collected from visits between November 2011 and September 2020, for a total of 344 visits from 102 type 2 SMA pediatric patients. None of the 102 patients died during the follow-up.

The mean follow-up was 2.81 years (SD: 3.71, range: 0–14): 44/102 (43%) had one visit only.

Demographic and clinical data of the patients are presented in Table 1.

**Table 1:** Demographic and clinical baseline data of the patients enrolled in the study

	**All**	**Longitudinal cohort**	**Statistical differences between the cohorts**
***N***	102	58	
***Sex, n (%)***			
*Male*	56 (54.90)	31 (53.45)	(*χ*(1) = 0.031, *p* = 0.859)
*Female*	46 (45.09)	27 (46.55)	
***Age at 1st visit (years), median (range)***	6.35 (1.1–19.24)	6.91 (1.9–19.24)	*p* = 1.000
***SMN2 copy number, n (%)***			
*1*	0 (0.00)	0 (0.00)	(*χ*(3) = 0.707, *p* = 0.871)
*2*	18 (17.64)	8 (13.79)	
*3*	56 (54.90)	31 (53.45)	
*4* +	3 (2.94)	2 (3.45)	
*Unknown*	25 (24.51)	17 (29.31)	
***SMA function at 1st visit, n (%)***			
*Non-sitter*	14 (13.72)	7 (12.07)	(*χ*(1) = 0.089, *p* = 0.765)
*Sitter*	88 (86.27)	51 (87.93)	
***BMI z-score at 1st visit, median (range)***	− 0.37 (− 16.68, 3.02)	0.02 (− 11.04–3.02)	*p* = 0.628
< *5 years*	− 1.17 (− 8.59, 2.82)	− 1.29 (− 4.39, 2.82)	*p* = 0.579
*5–12 years*	0.88 (− 7.75, 3.02)	0.82 (− 7.75, 3.02)	*p* = 0.871
> *13 years*	− 0.33 (− 19.1, 2.06)	− 0.33 (− 19.1, 2.06)	*p* = 0.657
***G-tube at 1st visit, n (%)***	1 (0.98)	0 (0.00)	(*χ*(1) = 0.572, *p* = 0.449)

**Fig. 1:**
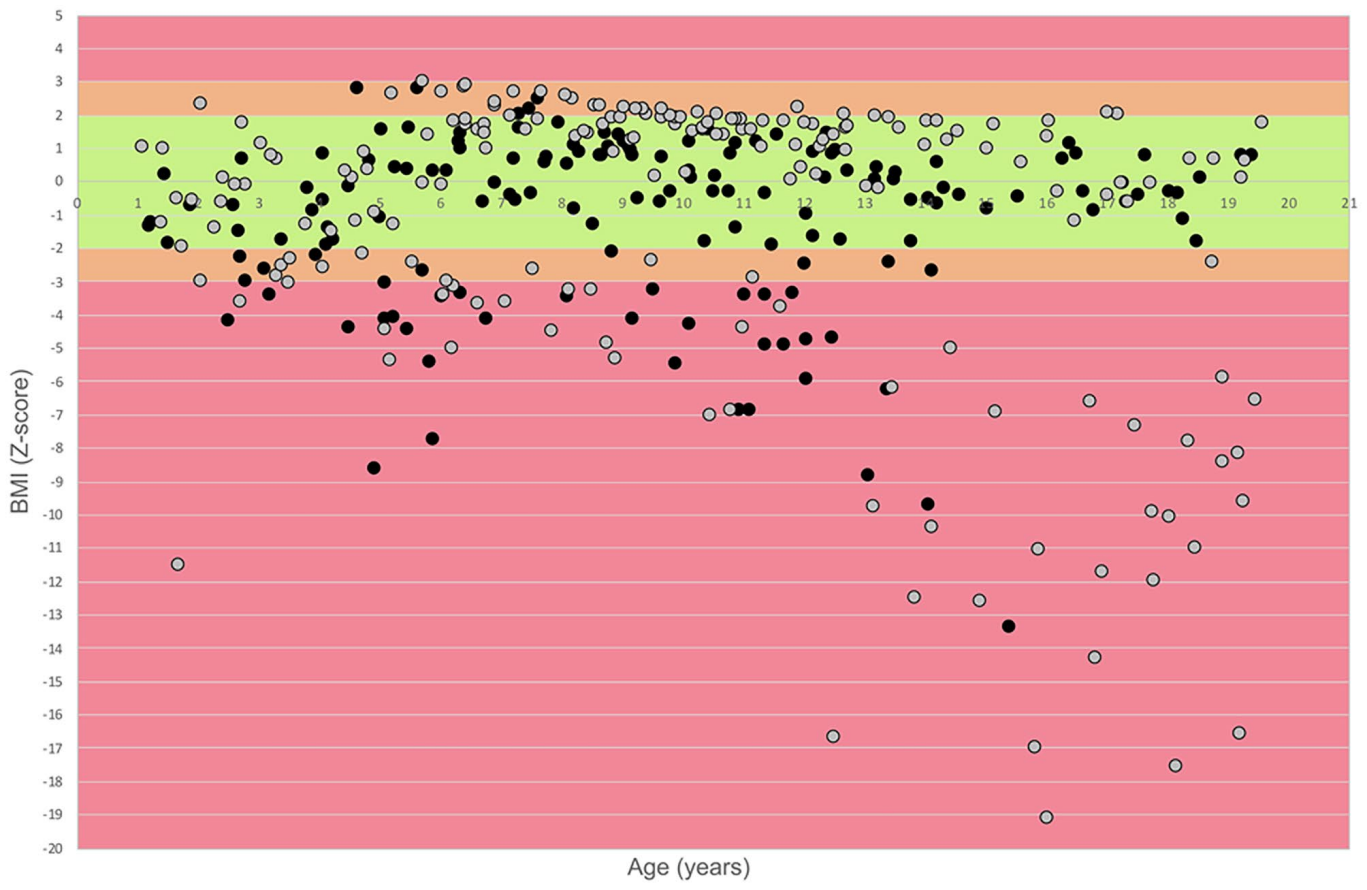
Cross-sectional distribution of BMI *z*-score according to age and gender. Key to figure = black dots: females, grey dots: male. Red bands: above or below ± 3SD, orange bands: above or below ± 2SD, green bands: between − 2 and 2SD

In the 344 visits, weight ranged between 3.90 and 83 kg, height between 51 and 176 cm, and the BMI between 8.4 and 31.6. The distribution of BMI/age *z*-scores of all 344 visits, in relation to age and gender, is shown in Fig. 1.

The graph shows relatively stable results below the age of 5 years and an increasing number of patients with BMI/age *z*-score <  − 2SD after the age of 5, with a wider range of BMI/age *z*-score after the age of 13.

A difference on the BMI/age *z*-score was found among the different age subgroups (< 5, 5–12, ≥ 13 years) (*p* < 0.001). Post hoc comparisons using the Dunn-Bonferroni correction indicated that the mean BMI/age *z*-score for the patients aged > 13 was significantly different than the patients aged 5–12 (*p* < 0.001). Patients aged 5–12 were significantly different than the patients aged < 5 (*p* = 0.001).

The BMI/age *z*-score was within normal range (± 2SD) in 215 of the 344 assessments (62%), >  + 2SD in 31/344 (9%), and <  − 2SD in the remaining 98/344 (28%). Twelve of the 344 visits were from 2 patients who had G-tube. The difference in mean BMI/age *z*-scores among the different age subgroups (< 5, 5–12, ≥ 13 years) was also significant when the analysis was performed in the patients with a *z*-score <  − 2SD (*p* < 0.001). Post hoc comparisons using the Dunn-Bonferroni correction indicated that the mean BMI/age *z*-score for the patients aged > 13 was significantly different from the patients aged < 5 (*p* < 0.001) and between 5 and 12 (*p* < 0.001). Table 2 reports data of BMI/age *z*-score distribution and mean (SD) for the age subgroups.

**Table 2:** BMI *z*-score distribution (*n*, %) and mean (SD) in the age subgroups

	** < − 2**	**± 2**	** > 2**
** < 5 years**			
*n* (%)	16 (26.23)	42 (68.85)	3 (4.92)
Mean (SD)	− 3.28 (1.56)	− 0.44 (1.00)	2.07 (0.93)
**5–12 years**			
*n* (%)	44 (26.04)	100 (59.17)	25 (14.79)
Mean (SD)	− 4.17 (1.36)	0.86 (0.95)	2.43 (0.30)
** > 13 years**			
*n* (%)	38 (33.33)	73 (64.04)	3 (2.63)
Mean (SD)	− 9.31 (4.45)	0.38 (1.00)	2.04 (0.03)

### BMI/age z-score and gender

Female patients had a higher percentage of assessments with BMI/age *z*-score ± 2SD compared to male patients (71% vs 56%) (*p* < 0.05), a lower but non-significant percentage of assessments with BMI/age *z*-score <  − 2SD (26% vs 39%), and a lower percentage of assessments with BMI/age *z*-score >  + 2SD (3% vs 14%) (*p* < 0.05) (*X*^2^ (2, *N* = 344) = 14.100, *p* < 0.001).

On the assessments performed after the age of 12 years, female had a higher percentage of BMI/age *z*-score ± 2SD compared to male patients (79% vs 53%) (*p* < 0.05) and a lower percentage of BMI/age *z*-score <  − 2SD compared to male patients (21% vs 42%) (*p* < 0.05). None of the female had a BMI/age *z*-score >  + 2SD (0% vs 5%) (*X*^2^ (2, *N* = 114) = 9.033, *p* = 0.011) (Fig. 1).

### BMI/age z-score and functional status

Patients who had lost the ability to sit (non-sitters) had a lower percentage of BMI/age *z*-score ± 2SD compared to sitters (39% vs 66%) (*p* < 0.05). There was also a higher percentage of BMI/age *z*-score <  − 2SD (61% vs 23%) (*p* < 0.05). None of the non-sitters had BMI/age *z*-score >  + 2SD (*X*^2^ (2, *N* = 344) = 31.789, *p* < 0.001).

### BMI/age z-score and NIV

Patients using NIV had a lower percentage of BMI/age *z*-score ± 2SD compared to those on spontaneous breathing (54% vs 68%) (*p* < 0.05), a higher percentage of BMI/age *z*-score <  − 2SD (42% vs 21%) (*p* < 0.05), and a lower percentage of BMI/age *z*-score >  + 2SD (5% vs 11%) (*p* < 0.05) (*X*^2^ (2, *N* = 344) = 18.202, *p* < 0.001).

### Longitudinal analysis

**Fig. 2:**
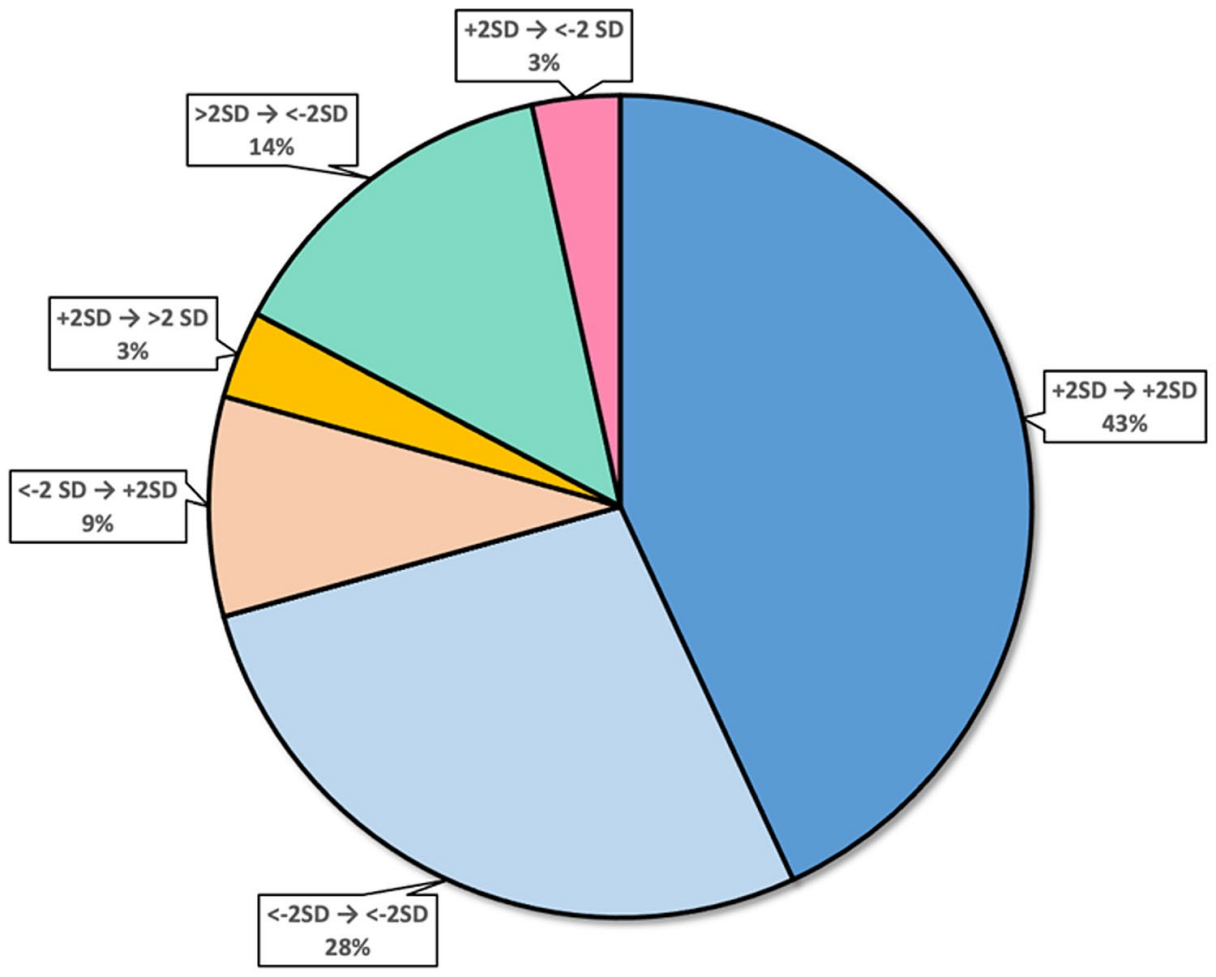
Percentage of patients with concordant or discordant BMI/age *z*-score bands (< − 2SD, ± 2SD, > 2SD) during follow-up

Fifty eight of 102 patients had more than one follow-up visit and were retained for the longitudinal analysis; the median number of visits was 4.0 (2–17), with a median follow-up of 3.87 years (0.2–14.0) (Fig. 2). No statistical difference was found between the baseline data in the whole cohort and the longitudinal cohort (Table 1). Details on patients’ inclusion can be found in supplementary Fig. 1.

Twenty-nine patients had baseline BMI/age *z*-score ± 2SD: 25 of the 29 remained ± 2SD, 2/29 decreased <  − 2SD, and 2/29 increased >  + 2SD; 21 patients had baseline BMI/age *z*-score <  − 2SD: 16 of the 21 remained <  − 2SD, 5/21 increased within ± 2SD, and 0/21 increased >  + 2SD; 8 patients had baseline BMI/age *z*-score >  + 2SD: 0 of the 8 remained > 2SD, 8/8 decreased to ± 2SD, and 0 decreased <  − 2SD.

**Fig. 3:**
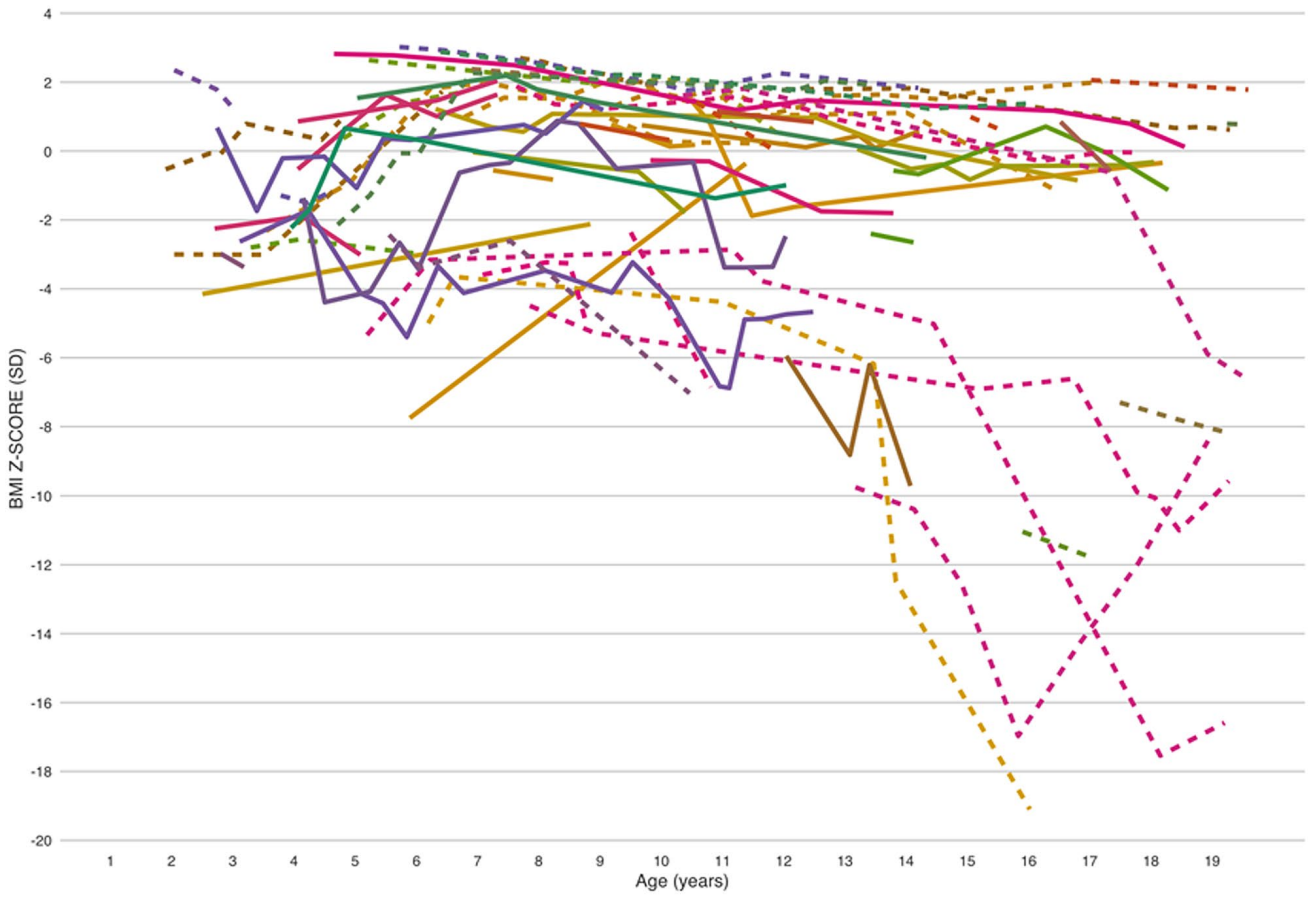
Individual trajectories by age and BMI/age *z*-score**.** Key to figure: dotted line = male patients, plain line = female patients

With one exception, all patients who, after the age of 13 years had BMI/age *z*-score <  − 5 SD, had their baseline BMI/age *z*-score already <  − 2SD, irrespective of the age at baseline. Figure 3 shows individual trajectories for the patients in whom more than 2 measurements were available.

### Mixed model analysis

Taking into account the 58 patients having more than one visit, the overall mean rate of weight BMI/age *z*-score decrease was 0.39/year. Only BMI/age *z*-score at baseline and gender were significantly contributing (*p* < 0.002 and < 0.001) to the changes (Table 1), with no significant impact of *SMN2* copy number, SMA function, non-invasive ventilation, nutritional status, and scoliosis surgery on rate of progression (*p* for interaction between time and other variables > 0.05).

## Discussion

Both the first and the revised version of the care recommendations for SMA [32, 47] promote close monitoring of nutritional aspects and highlight the need to perform regular anthropometric assessments in combination with the evaluation of other factors (swallowing, gastroesophageal reflux, etc.) that may contribute to weight gain. Little has been reported on nutritional issues in type 2 children and adults [20, 25, 26, 30, 31, 48, 49]. Most studies have focused on BMI, as this is easy to define and can always be assessed as part of routine clinical assessments. The choice of BMI is also driven by the increasing evidence of its predictive value for clinical disease outcomes in children[50, 51].

The few available studies report a relatively high percentage of patients with BMI/age *z*-score <  − 2SD but the rate or severity of the reported findings is not univocal [30, 31]. The identification of patients with nutritional issues is further complicated by the poor representation of lean body mass that makes interpretation of the standard growth chart references difficult [48].

In our study 31 patients had at least one assessment with a BMI/age *z*-score <  − 2SD that was present in 98 of the 344 measurements (28%). The number of patients with BMI/age *z*-score >  + 2SD was in contrast relatively small and this was mainly observed in boys below the age of 13 years.

The analysis of longitudinal changes allowed us further considerations. It is of note that the number of patients who had concordant results, remaining in the same subgroup (± 2SD, <  − 2S, or >  + 2SD), was very high (82%). Half of the patients remained within ± 2SD while the number of patients who went from ± 2 to <  − 2SD was relatively small.

The lowest BMI/age *z*-scores were mainly observed in patients older than 12 years in whom the BMI/age *z*-score values were often already <  − 2SD at first assessment, irrespective of the age when the first assessment was performed. This occurred more frequently in male patients. At the other end of the spectrum, a number of boys who were >  + 2SD between the age of 5 and 12 shifted to ± 2SD when approaching puberty.

In this paper we were also interested in better understanding possible factors that may be associated with low BMI. Unfortunately, our registries did not collect information on chewing and masticatory strength that are known to contribute to failure to thrive [20, 27]. When we considered individual assessments the risk of having a low BMI was higher in more severely affected patients, such as the non-sitters and those using non-invasive ventilation.

A multivariate analysis exploring the impact of possible variables on the changes in patients with longitudinal assessments showed that BMI/age *z*-score at baseline and gender were significantly contributing (*p* < 0.002 and < 0.001) to the changes while other variables, such as *SMN2* copy number, functional status, non-invasive ventilation, and nutritional status, were not. As in 25% of the patients the number of SMN2 copies was not available because this was not systematically performed until recently, the lack of significance for this variable should be interpreted with caution.

It is of note that in a number of assessments the BMI/age *z*-score was between − 2 and − 3SD. These findings however were calculated on standard growth charts that may be not entirely appropriate for SMA patients in whom the poor weight may be related to the poor representation of muscles and lean mass [48, 52]. While lower BMI are undoubtedly related to poor weight gain, values between − 2 and − 3SD should be further assessed with techniques exploring fat mass/fat free mass ratio (DXA, BIA) or using plicometry.

At variance with other studies [30], we had a low number of patients who underwent gastrostomy, despite the low BMI. Only two of the patients had a history of swallowing problems, and/or aspiration pneumonia, and they both underwent gastrostomy. In all the others, after introduction of supplements and dietary recommendations, the possibility of a PEG was discussed, but as the majority of the cases with very low BMI were in their teens, they would not accept this option for a variety of reasons, including issues related to body image.

In conclusion our results confirm that low BMI/age *z*-score is a frequent feature in type 2 SMA. Although our cross-sectional and longitudinal data could not be directly compared because of the difference in the sample size, both showed the reduction of BMI with increasing age. Our findings are within the range of BMI/age *z*-score reported in the literature but the number of low BMI/age *z*-scores was smaller compared to other studies. Possible country or cultural related differences should be further explored.

In our study patients with a low BMI/age *z*-score at baseline were at higher risk of developing further reduction, highlighting the need of regular surveillance of anthropometric factors. Gender also appeared to be another significant risk factor as male patients showed more obvious changes in weight at both extremes of the range. Further studies, including assessments of chewing and swallowing and other methods aimed at assessing lean and fat body mass, will help to better stratify the type 2 cohort and achieve a better understanding of the possible mechanisms underlying nutritional issues.

## Supplementary Information

Below is the link to the electronic supplementary material.

Supplementary file1 (TIF 119 KB)

Supplementary file2 (DOCX 44 KB)

## Data Availability

All data are within manuscript ; data are available upon reasonable request to the corresponding author.

## Abbreviations

BMI: Body mass index
NIV: Non-invasive ventilation
SMA: Spinal muscular atrophy
SMN: Survival motor neuron

## Code availability

N/A.

## References

[1] D'Amico A, Mercuri E, Tiziano FD, Bertini E (2011). Spinal muscular atrophy. Orphanet J Rare Dis.

[2] Finkel RS, Mercuri E, Darras BT, Connolly AM, Kuntz NL, Kirschner J, Chiriboga CA, Saito K, Servais L, Tizzano E, Topaloglu H, Tulinius M, Montes J, Glanzman AM, Bishop K, Zhong ZJ, Gheuens S, Bennett CF, Schneider E, Farwell W, De Vivo DC, Group ES (2017). Nusinersen versus sham control in infantile-onset spinal muscular atrophy. N Engl J Med.

[3] Mercuri E, Darras BT, Chiriboga CA, Day JW, Campbell C, Connolly AM, Iannaccone ST, Kirschner J, Kuntz NL, Saito K, Shieh PB, Tulinius M, Mazzone ES, Montes J, Bishop KM, Yang Q, Foster R, Gheuens S, Bennett CF, Farwell W, Schneider E, De Vivo DC, Finkel RS, Group CS (2018). Nusinersen versus sham control in later-onset spinal muscular atrophy. N Engl J Med.

[4] Baranello G, Darras BT, Day JW, Deconinck N, Klein A, Masson R, Mercuri E, Rose K, El-Khairi M, Gerber M, Gorni K, Khwaja O, Kletzl H, Scalco RS, Seabrook T, Fontoura P, Servais L, Group FW (2021). Risdiplam in type 1 spinal muscular atrophy. N Engl J Med.

[5] Mendell JR, Al-Zaidy S, Shell R, Arnold WD, Rodino-Klapac LR, Prior TW, Lowes L (2017). Single-dose gene-replacement therapy for spinal muscular atrophy. N Engl J Med.

[6] Hagenacker T, Wurster CD, Gunther R, Schreiber-Katz O, Osmanovic A, Petri S, Weiler M (2020). Nusinersen in adults with 5q spinal muscular atrophy: a non-interventional, multicentre, observational cohort study. Lancet Neurol.

[7] Pechmann A, Langer T, Wider S, Kirschner J (2018). Single-center experience with intrathecal administration of Nusinersen in children with spinal muscular atrophy type 1. Eur J Paediatr Neurol.

[8] Coratti G, Pane M, Lucibello S, Pera MC, Pasternak A, Montes J, Sansone VA et al (2021) Age related treatment effect in type Ii spinal muscular atrophy pediatric patients treated with nusinersen. Neuromuscul Disord 31(7):596–602 10.1016/j.nmd.2021.03.012 34099377

[9] Aragon-Gawinska K, Seferian AM, Daron A, Gargaun E, Vuillerot C, Cances C, Ropars J, Chouchane M, Cuppen I, Hughes I, Illingworth M, Marini-Bettolo C, Rambaud J, Taytard J, Annoussamy M, Scoto M, Gidaro T, Servais L (2018). Nusinersen in patients older than 7 months with spinal muscular atrophy type 1: a cohort study. Neurology.

[10] Szabo L, Gergely A, Jakus R, Fogarasi A, Grosz Z, Molnar MJ, Andor I, Schulcz O, Goschler A, Medveczky E, Czovek D, Herczegfalvi A (2020). Efficacy of nusinersen in type 1, 2 and 3 spinal muscular atrophy: real world data from Hungarian patients. Eur J Paediatr Neurol.

[11] Coratti G, Lucibello S, Pera MC, Duong T, Muni Lofra R, Civitello M, D'Amico A, Goemans N, Darras BT, Bruno C, Sansone VA, Day J, Nascimento Osorio A, Muntoni F, Montes J, Sframeli M, Finkel R, Mercuri E, group  I (2020). Gain and loss of abilities in type II SMA: a 12-month natural history study. Neuromuscul Disord.

[12] Coratti G, Messina S, Lucibello S, Pera MC, Montes J, Pasternak A, Bovis F (2020). Clinical variability in spinal muscular atrophy type III. Ann Neurol.

[13] Coratti G, Pera MC, Lucibello S, Montes J, Pasternak A, Mayhew A, Glanzman AM, Young SD, Pane M, Scoto M, Messina S, Goemans N, Osorio AN, Pedemonte M, Sansone V, Bertini E, De Vivo DC, Finkel R, Muntoni F, Mercuri E, group I, collaborators,  (2020). Age and baseline values predict 12 and 24-month functional changes in type 2 SMA. Neuromuscul Disord.

[14] De Sanctis R, Pane M, Coratti G, Palermo C, Leone D, Pera MC, Abiusi E, Fiori S, Forcina N, Fanelli L, Lucibello S, Mazzone ES, Tiziano FD, Mercuri E (2018). Clinical phenotypes and trajectories of disease progression in type 1 spinal muscular atrophy. Neuromuscul Disord.

[15] Mercuri E, Lucibello S, Pera MC, Carnicella S, Coratti G, de Sanctis R, Messina S, Mazzone E, Forcina N, Fanelli L, Norcia G, Antonaci L, Frongia AL, Pane M (2019). Long-term progression in type II spinal muscular atrophy: a retrospective observational study. Neurology.

[16] Annoussamy M, Seferian AM, Daron A, Pereon Y, Cances C, Vuillerot C, De Waele L, Laugel V, Schara U, Gidaro T, Lilien C, Hogrel JY, Carlier P, Fournier E, Lowes L, Gorni K, Ly-Le Moal M, Hellbach N, Seabrook T, Czech C, Hermosilla R, Servais L, NatHis  SMAsg (2021). Natural history of type 2 and 3 spinal muscular atrophy: 2-year NatHis-SMA study. Ann Clin Transl Neurol.

[17] Trucco F, Ridout D, Scoto M, Coratti G, Main ML, Muni Lofra R, Mayhew AG (2021). Respiratory trajectories in type 2 and 3 spinal muscular atrophy in the iSMAC cohort study. Neurology.

[18] Finkel RS, McDermott MP, Kaufmann P, Darras BT, Chung WK, Sproule DM, Kang PB, Foley AR, Yang ML, Martens WB, Oskoui M, Glanzman AM, Flickinger J, Montes J, Dunaway S, O'Hagen J, Quigley J, Riley S, Benton M, Ryan PA, Montgomery M, Marra J, Gooch C, De Vivo DC (2014). Observational study of spinal muscular atrophy type I and implications for clinical trials. Neurology.

[19] Kolb SJ, Coffey CS, Yankey JW, Krosschell K, Arnold WD, Rutkove SB, Swoboda KJ (2017). Natural history of infantile-onset spinal muscular atrophy. Ann Neurol.

[20] Messina S, Pane M, De Rose P, Vasta I, Sorleti D, Aloysius A, Sciarra F, Mangiola F, Kinali M, Bertini E, Mercuri E (2008). Feeding problems and malnutrition in spinal muscular atrophy type II. Neuromuscul Disord.

[21] van den Engel-Hoek L, de Groot IJ, de Swart BJ, Erasmus CE (2015). Feeding and swallowing disorders in pediatric neuromuscular diseases: an overview. J Neuromuscul Dis.

[22] Wadman RI, van Bruggen HW, Witkamp TD, Sparreboom-Kalaykova SI, Stam M, van den Berg LH, Steenks MH, van der Pol WL (2014). Bulbar muscle MRI changes in patients with SMA with reduced mouth opening and dysphagia. Neurology.

[23] Chen YS, Shih HH, Chen TH, Kuo CH, Jong YJ (2012) Prevalence and risk factors for feeding and swallowing difficulties in spinal muscular atrophy types II and III. J Pediatr 160:447–451 e441 10.1016/j.jpeds.2011.08.016 21924737

[24] Chabanon A, Seferian AM, Daron A, Pereon Y, Cances C, Vuillerot C, De Waele L, Cuisset JM, Laugel V, Schara U, Gidaro T, Gilabert S, Hogrel JY, Baudin PY, Carlier P, Fournier E, Lowes LP, Hellbach N, Seabrook T, Toledano E, Annoussamy M, Servais L, NatHis SMAsg (2018) Prospective and longitudinal natural history study of patients with type 2 and 3 spinal muscular atrophy: baseline data NatHis-SMA study. PLoS One 13:e0201004 10.1371/journal.pone.0201004 PMC6062049 30048507

[25] Sproule DM, Montes J, Dunaway S, Montgomery M, Battista V, Koenigsberger D, Martens B, Shen W, Punyanitya M, Benton M, Butler H, Caracciolo J, Mercuri E, Finkel R, Darras B, De Vivo DC, Kaufmann P (2010). Adiposity is increased among high-functioning, non-ambulatory patients with spinal muscular atrophy. Neuromuscul Disord.

[26] Sproule DM, Montes J, Montgomery M, Battista V, Koenigsberger D, Shen W, Punyanitya M, De Vivo DC, Kaufmann P (2009). Increased fat mass and high incidence of overweight despite low body mass index in patients with spinal muscular atrophy. Neuromuscul Disord.

[27] van Bruggen HW, van den Engel-Hoek L, van der Pol WL, de Wijer A, de Groot IJ, Steenks MH (2011). Impaired mandibular function in spinal muscular atrophy type II: need for early recognition. J Child Neurol.

[28] van Bruggen HW, Wadman RI, Bronkhorst EM, Leeuw M, Creugers N, Kalaykova SI, van der Pol WL, Steenks MH (2016). Mandibular dysfunction as a reflection of bulbar involvement in SMA type 2 and 3. Neurology.

[29] Mehta NM, Newman H, Tarrant S, Graham RJ (2016). Nutritional status and nutrient intake challenges in children with spinal muscular atrophy. Pediatr Neurol.

[30] Wadman RI, De Amicis R, Brusa C, Battezzati A, Bertoli S, Davis T, Main M, Manzur A, Mastella C, Munot P, Imbrigiotta N, Schottlaender L, Sarkozy A, Trucco F, Baranello G, Scoto M, Muntoni F (2021). Feeding difficulties in children and adolescents with spinal muscular atrophy type 2. Neuromuscul Disord.

[31] De Amicis R, Baranello G, Foppiani A, Leone A, Battezzati A, Bedogni G, Ravella S, Giaquinto E, Mastella C, Agosto C, Bertini E, D'Amico A, Pedemonte M, Bruno C, Wells JC, Fewtrell M, Bertoli S (2021). Growth patterns in children with spinal muscular atrophy. Orphanet J Rare Dis.

[32] Mercuri E, Finkel RS, Muntoni F, Wirth B, Montes J, Main M, Mazzone ES, Vitale M, Snyder B, Quijano-Roy S, Bertini E, Davis RH, Meyer OH, Simonds AK, Schroth MK, Graham RJ, Kirschner J, Iannaccone ST, Crawford TO, Woods S, Qian Y, Sejersen T, Group SMAC (2018). Diagnosis and management of spinal muscular atrophy: Part 1: Recommendations for diagnosis, rehabilitation, orthopedic and nutritional care. Neuromuscul Disord.

[33] Mercuri E, Finkel R, Scoto M, Hall S, Eaton S, Rashid A, Balashkina J, Coratti G, Pera MC, Samsuddin S, Civitello M, Muntoni F, i SG (2019). Development of an academic disease registry for spinal muscular atrophy. Neuromuscul Disord.

[34] Gauld LM, Kappers J, Carlin JB, Robertson CF (2004). Height prediction from ulna length. Dev Med Child Neurol.

[35] Madden AM, Tsikoura T, Stott DJ (2012). The estimation of body height from ulna length in healthy adults from different ethnic groups. J Hum Nutr Diet.

[36] Gauld LM, Keeling LA, Shackleton CE, Sly PD (2014). Forced oscillation technique in spinal muscular atrophy. Chest.

[37] Bertoli S, Foppiani A, De Amicis R, Leone A, Mastella C, Bassano M, Giaquinto E, Baranello G, Battezzati A (2019). Anthropometric measurement standardization for a multicenter nutrition survey in children with spinal muscular atrophy. Eur J Clin Nutr.

[38] Grummer-Strawn LM, Reinold C, Krebs NF, Centers for Disease C, Prevention (2010). Use of World Health Organization and CDC growth charts for children aged 0–59 months in the United States. MMWR Recomm Rep.

[39] de Onis M, Garza C, Onyango AW, Rolland-Cachera MF, le Comite de nutrition de la Societe francaise de p (2009). WHO growth standards for infants and young children. Arch Pediatr.

[40] Kuczmarski RJ, Ogden CL, Guo SS, Grummer-Strawn LM, Flegal KM, Mei Z, Wei R, Curtin LR, Roche AF, Johnson CL (2002). 2000 CDC Growth Charts for the United States: methods and development. Vital Health Stat.

[41] Barlow SE, Expert C (2007). Expert committee recommendations regarding the prevention, assessment, and treatment of child and adolescent overweight and obesity: summary report. Pediatrics.

[42] Cote AT, Harris KC, Panagiotopoulos C, Sandor GG, Devlin AM (2013). Childhood obesity and cardiovascular dysfunction. J Am Coll Cardiol.

[43] Coratti G, Pane M, Lucibello S, Pera MC, Pasternak A, Montes J, Sansone VA (2021). Age related treatment effect in type II Spinal Muscular Atrophy pediatric patients treated with nusinersen. Neuromuscul Disord.

[44] Pera MC, Coratti G, Mazzone ES, Montes J, Scoto M, De Sanctis R, Main M, Mayhew A, Muni Lofra R, Dunaway Young S, Glanzman AM, Duong T, Pasternak A, Ramsey D, Darras B, Day JW, Finkel RS, De Vivo DC, Sormani MP, Bovis F, Straub V, Muntoni F, Pane M, Mercuri E, i SCG (2019). Revised upper limb module for spinal muscular atrophy: 12 month changes. Muscle Nerve.

[45] O'Hagen JM, Glanzman AM, McDermott MP, Ryan PA, Flickinger J, Quigley J, Riley S, Sanborn E, Irvine C, Martens WB, Annis C, Tawil R, Oskoui M, Darras BT, Finkel RS, De Vivo DC (2007). An expanded version of the Hammersmith Functional Motor Scale for SMA II and III patients. Neuromuscul Disord.

[46] Coratti G, Pera MC, Lucibello S, Montes J, Pasternak A, Mayhew A, Glanzman A, Dunaway Young S, Pane M, Scoto M, Messina S, Goemans N, Nascimiento O, Pedemonte M, Sansone V, Bertini E, De Vivo DC, Finkel R, Muntoni FEM (2020) Age and baseline values predict 12 and 24-month functional changes in type 2 SMA. Neuromuscul Disord 30(9):756–764 10.1016/j.nmd.2020.07.005 32900576

[47] Wang CH, Finkel RS, Bertini ES, Schroth M, Simonds A, Wong B, Aloysius A, Morrison L, Main M, Crawford TO, Trela A (2007). Consensus statement for standard of care in spinal muscular atrophy. J Child Neurol.

[48] Bertoli S, De Amicis R, Mastella C, Pieri G, Giaquinto E, Battezzati A, Leone A, Baranello G (2017). Spinal Muscular Atrophy, types I and II: what are the differences in body composition and resting energy expenditure?. Clin Nutr.

[49] Poruk KE, Davis RH, Smart AL, Chisum BS, Lasalle BA, Chan GM, Gill G, Reyna SP, Swoboda KJ (2012). Observational study of caloric and nutrient intake, bone density, and body composition in infants and children with spinal muscular atrophy type I. Neuromuscul Disord.

[50] Trandafir LM, Cojocaru E, Moscalu M, Leon Constantin MM, Miron I, Mastaleru A, Teslariu O, Datcu ME, Fotea S, Frasinariu O (2021) Predictive markers of early cardiovascular impairment and insulin resistance in obese pediatric patients. Diagnostics (Basel) 11 10.3390/diagnostics11040735 PMC8074748 33924229

[51] Pischon T, Boeing H, Hoffmann K, Bergmann M, Schulze MB, Overvad K, van der Schouw YT (2008). General and abdominal adiposity and risk of death in Europe. N Engl J Med.

[52] Foppiani A, De Amicis R, Leone A, Ravella S, Bedogni G, Battezzati A, D'Amico A, Bertini E, Pedemonte M, Bruno C, Agosto C, Mastella C, Giaquinto E, Masson R, Baranello G, Bertoli S (2021). Predictive fat mass equations for spinal muscular atrophy type I children: development and internal validation. Clin Nutr.

