# Supplementary figures and images for: Body mass index in type 2 spinal muscular atrophy: a longitudinal study
Source: Eur J Pediatr. 2022 Jan 19;181(5):1923–32. doi: 10.1007/s00431-021-04325-3 (PMC9056453; doi:10.1007/s00431-021-04325-3)

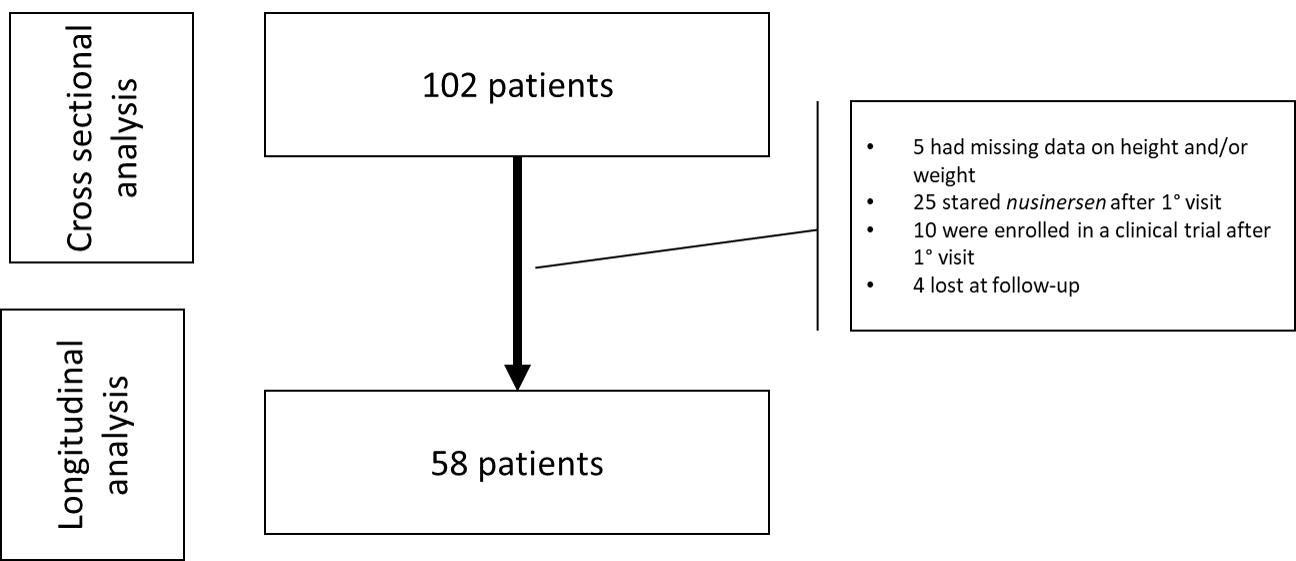

Supplement: Supplementary file 1 — Supplementary file1 (TIF 119 KB) [file 431_2021_4325_MOESM1_ESM.tif]
