# Supplementary material for: Body mass index in type 2 spinal muscular atrophy: a longitudinal study
Source: Eur J Pediatr. 2022 Jan 19;181(5):1923–32. doi: 10.1007/s00431-021-04325-3 (PMC9056453; doi:10.1007/s00431-021-04325-3)
Supplement: Supplementary file 2 — Supplementary file2 (DOCX 44 KB) [file 431_2021_4325_MOESM2_ESM.docx]

**Missing data analysis**

*Descriptive statistics on data*

| **Statistics** | | | |
| --- | --- | --- | --- |
|  | | Height | Weight |
| N | Valid | 353 | 653 |
|  | Missing | 300 | 0 |
| Mean | | 113,5 | 27,9 |
| Mean Std. Deviation | | 2,8 | ,6 |
| Median | | 130,0 | 24,0 |
| Mode | | 140,0 | 12,0 |
| St. Deviation | | 52,4 | 15,8 |
| Viariance | | 2750,2 | 251,3 |
| Skweness | | -1,4 | ,9 |
| Std. Error of Skewnss | | ,1 | ,1 |
| Kurtosis | | ,6 | ,3 |
| Std. Error of Kurtosis | | ,3 | ,2 |

| **missingdata** | | | | | |
| --- | --- | --- | --- | --- | --- |
|  | | Frequency | Percent | Valid Percent | Cumulative percent |
| Valid | ,00 | 353 | 54,1 | 54,1 | 54,1 |
|  | 1,00 | 300 | 45,9 | 45,9 | 100,0 |
|  | Total | 653 | 100,0 | 100,0 |  |

*Statistical analysis*

| **Group statistics** | | | | | |
| --- | --- | --- | --- | --- | --- |
|  | height_missing | N | Mean | Std. Deviation | Std. Error Mean |
| Age | ,00 | 300 | 7,6 | 4,5 | ,3 |
|  | 1,00 | 353 | 9,80 | 4,8 | ,3 |
| height | ,00 | 0^a^ | . | . | . |
|  | 1,00 | 353 | 113,50 | 52,4 | 2,8 |

| **Independent Samples Tests** | | | | | | | | | | | |
| --- | --- | --- | --- | --- | --- | --- | --- | --- | --- | --- | --- |
|  | | Levene’s test | | t-test for Equality of Means | | | | | | | |
|  |  | F | Sign. | t | gl | Signifincance (2-sided) | Difference from mean | Difference std. error | C.I. 95% | |  |
|  |  |  |  |  |  |  |  |  | Inferior | Superior |  |
| Age | Equal variances assumed | 2,002 | ,158 | -6,010 | 651 | ,000 | -2,19893 | ,36585 | -2,91732 | -1,48054 |  |
|  | Equal variances not assumed |  |  | -6,048 | 646,159 | ,000 | -2,19893 | ,36359 | -2,91289 | -1,48498 |  |

**GENDER * height_missing**

| **Contingency table** | | | | |
| --- | --- | --- | --- | --- |
| Count | | | | |
|  | | height_missing | | Total |
|  |  | ,00 | 1,00 |  |
| GENDER | Female | 147 | 163 | 310 |
|  | Male | 153 | 190 | 343 |
| Total | | 300 | 353 | 653 |

| **Chi-Square Tests** | | | | | |
| --- | --- | --- | --- | --- | --- |
|  | Value | df | Asymptotic Signifincance (2-sided) | Exact Sig. (2-sided) | Exact Sig (1-sided) |
| Pearson Chi-Square | ,519^a^ | 1 | ,471 |  |  |
| Continuity correction ^b^ | ,412 | 1 | ,521 |  |  |
| Likelihood Ratio | ,519 | 1 | ,471 |  |  |
| Fisher’s Exact Test |  |  |  | ,480 | ,261 |
| N of Valid Cases | 653 |  |  |  |  |

**SMAFUNCTION * height_missing**

| **Contingency table** | | | | |
| --- | --- | --- | --- | --- |
| Count | | | | |
|  | | height_missing | | Total |
|  |  | ,00 | 1,00 |  |
| SMAFUNCTION | Non sitter | 27 | 49 | 76 |
|  | Sitter | 273 | 304 | 577 |
| Total | | 300 | 353 | 653 |

| **Chi-Square Tests** | | | | | |
| --- | --- | --- | --- | --- | --- |
|  | Value | df | Asymptotic Signifincance (2-sided) | Exact Sig. (2-sided) | Exact Sig (1-sided) |
| Pearson Chi-Square | 3,757^a^ | 1 | ,053 |  |  |
| Continuity correction ^b^ | 3,297 | 1 | ,069 |  |  |
| Likelihood Ratio | 3,820 | 1 | ,051 |  |  |
| Fisher’s Exact Test |  |  |  | ,066 | ,034 |
| N of Valid Cases | 653 |  |  |  |  |

*Conclusions*

Since no statistical significance was found between missing data and population characteristics, we can assume that these are completely at random.
